# A Unified Multidimensional Benchmark and Multi-Dataset Evaluation of YOLO-Based Models for Remote Sensing Building Instance Segmentation

**DOI:** 10.3390/s26123686

**Published:** 2026-06-09

**Authors:** Zhengsheng Chen, Junjie Xu, Dongdong Guan, Xiaolong Zheng, Yujie Li

**Affiliations:** PLA Rocket Force University of Engineering, Xi’an 710025, China; xjjdvq@126.com (J.X.); gdd@whu.edu.cn (D.G.); xxgmzz@163.com (X.Z.); lyjrfue@126.com (Y.L.)

**Keywords:** YOLO, building segmentation, remote sensing, model evaluation

## Abstract

**Highlights:**

**What are the main findings?**

- A unified multidimensional evaluation framework is proposed for systematic benchmarking of YOLO-based and representative instance segmentation models in remote sensing building extraction.
- YOLO-based models achieved competitive performance in the evaluated setting, with YOLOv11x-seg delivering the highest accuracy and YOLOv11m-seg achieving the best balance between accuracy, efficiency, and model complexity.

**What are the implications of the main findings?**

- The study provides quantitative and practical reference evidence for model selection under different application requirements, including high-precision analysis and real-time deployment.
- The results show favorable performance under the tested dataset setting of single-stage YOLO-seg frameworks over two-stage and Transformer-based methods in complex remote sensing scenarios, offering insights for future model design and optimization.

**Abstract:**

Building instance segmentation in remote sensing imagery supports applications such as urban management, disaster assessment, 3D urban modeling, and land-cover monitoring. However, variations in building scale, dense spatial distribution, complex background textures, shadows, and occlusions make it difficult to balance segmentation accuracy, boundary recovery, inference efficiency, and deployment cost. This study establishes a unified multidimensional benchmark for remote sensing building instance segmentation. The primary benchmark evaluates mask-predicting instance segmentation models, including YOLOv8-seg, YOLOv11-seg, YOLO26-seg, and Mask R-CNN, under consistent training and evaluation settings. RT-DETR-l and RT-DETR-x are retained only as auxiliary detection-only Transformer baselines because they do not output instance masks in the implemented setting. The benchmark covers bounding-box detection, mask-based segmentation, inference efficiency, model complexity, training behavior, and qualitative visualization. To assess cross-dataset transferability and degradation-specific robustness beyond a single dataset, we further conduct zero-shot WHU-to-Inria testing, independent Inria training/testing with different initialization strategies, and controlled degradation tests involving shadow/occlusion and Gaussian blur. Results on WHU and Inria show that high-capacity YOLO-seg models are competitive among the evaluated mask-predicting models. Under the current experimental settings, YOLOv11x-seg achieves the highest or near-highest mask-based accuracy, whereas YOLOv11m-seg provides a favorable balance between accuracy, speed, and complexity. The zero-shot WHU-to-Inria test reveals a clear domain shift, while the Inria in-domain experiments indicate that high-capacity YOLO-seg models recover competitive performance after target-domain training. The controlled degradation tests show a smaller performance drop under shadow/occlusion than under Gaussian blur for YOLOv11x-seg. These findings provide benchmark-specific evidence for selecting remote sensing building instance segmentation models under accuracy-oriented and efficiency-oriented deployment requirements.

## 1. Introduction

The accurate identification and segmentation of building objects in remote sensing images provide critical technical support for refined urban planning, disaster assessment, 3D urban modeling, land-cover monitoring, and the intelligent interpretation of Earth observation data. However, building extraction from high-resolution remote sensing imagery remains challenging because of variations in sensor imaging conditions, complex geographic environments, and the diverse morphology of buildings. In dense urban areas, buildings often show large scale variations, irregular shapes, complex spatial arrangements, and high inter-instance adjacency. In addition, shadows, vegetation, roads, occlusions, and background textures may blur building boundaries and reduce the separability between buildings and surrounding objects. These factors make it difficult for segmentation models to simultaneously achieve accurate localization, complete boundary recovery, high inference efficiency, and stable robustness.

Traditional machine learning methods usually rely on handcrafted features, such as spectral, textural, and geometric descriptors. Although these methods are interpretable, their generalization ability is limited in complex urban scenes. In recent years, deep learning has significantly advanced remote sensing building segmentation. CNN-based encoder–decoder architectures, such as FCN, U-Net, and DeepLab variants, have been widely used for pixel-level building extraction because they can integrate multi-scale semantic and spatial information. However, semantic segmentation methods generally predict category-level masks and do not explicitly distinguish adjacent building instances, which limits their applicability in dense building scenes [1,2]. To improve boundary recovery and contextual modeling, existing studies have introduced attention mechanisms, feature pyramid structures, edge-aware modules, and Transformer-based architectures. These strategies improve feature representation to some extent, but challenges remain in separating adjacent buildings, recovering fine boundaries, and maintaining robustness under shadows, occlusions, and complex backgrounds.

For instance-level building segmentation, existing methods can be broadly divided into two-stage, single-stage, and Transformer-based paradigms. Two-stage methods, represented by Mask R-CNN, first generate candidate regions and then perform classification, bounding-box regression, and mask prediction [3,4]. They are effective for instance-level localization and mask refinement, but their region proposal and RoI-based processing usually introduce relatively high computational cost. In contrast, single-stage YOLO-based segmentation models predict object locations and instance masks in an end-to-end manner, providing a favorable balance between accuracy and inference efficiency. This makes them attractive for large-scale remote sensing image interpretation and real-time deployment. Transformer-based detectors, such as RT-DETR, further introduce global context modeling and set prediction mechanisms, offering a complementary detection paradigm. Nevertheless, when such models do not include a dedicated mask prediction head, their role in instance segmentation benchmarks should be limited to bounding-box-level detection analysis rather than mask-level comparison.

Although considerable progress has been made in remote sensing building segmentation, existing studies usually focus on improving a specific model, designing a dedicated module, or validating performance on a limited dataset [5,6,7,8,9,10]. A unified and reproducible benchmark for comparing multiple YOLO-based instance segmentation models with representative two-stage and Transformer-based baselines remains insufficient. In particular, few studies jointly evaluate detection accuracy, mask quality, inference efficiency, model complexity, training behavior, cross-dataset transferability, and degradation-specific robustness under consistent experimental settings. This makes it difficult to provide practical evidence for selecting appropriate models under different application requirements, such as accuracy-oriented mapping, real-time interpretation, or resource-constrained deployment [11,12,13,14,15,16,17].

To address this gap, this study establishes a unified multidimensional benchmark for remote sensing building instance segmentation. The primary benchmark evaluates mask-predicting instance segmentation models, including YOLOv8-seg, YOLOv11-seg, YOLO26-seg, and Mask R-CNN, under consistent training and evaluation protocols. RT-DETR-l and RT-DETR-x are retained only as auxiliary detection-only Transformer baselines because they do not output instance masks in the implemented setting. The benchmark covers bounding-box detection, mask-based segmentation, inference efficiency, model complexity, training behavior, and qualitative visualization. In addition, to assess generalization and robustness beyond a single dataset, this study conducts zero-shot WHU-to-Inria testing, independent Inria training/testing with different initialization strategies, and controlled degradation tests involving shadow/occlusion and Gaussian blur.

The main contributions of this paper are as follows:

1. This study constructs a unified and reproducible benchmark for remote sensing building instance segmentation. It does not propose a new network architecture, loss function, dataset, or segmentation module. Instead, it provides systematic comparative evidence for representative YOLO-based segmentation models and baseline methods under consistent training and evaluation settings.
2. The benchmark evaluates representative mask-predicting instance segmentation models, including YOLOv8-seg, YOLOv11-seg, YOLO26-seg, and Mask R-CNN, from multiple perspectives, including detection accuracy, mask quality, inference efficiency, model complexity, training behavior, and qualitative visualization. RT-DETR is retained only as a detection-only baseline for bounding-box-level analysis.
3. To strengthen the robustness evidence beyond a single dataset, this study further conducts three complementary evaluations: zero-shot WHU-to-Inria testing, independent training and testing on the Inria dataset with different initialization strategies, and controlled degradation tests involving shadow/occlusion and Gaussian blur. These experiments provide additional evidence for cross-dataset generalization, initialization sensitivity, and degradation-specific robustness.

The remainder of this paper is organized as follows. Section 2 introduces the unified benchmark framework, evaluated model groups, and task-oriented model-suitability analysis. Section 3 presents the experimental setup, datasets, evaluation metrics, implementation details, and experimental results. Section 4 discusses the main findings, limitations, and future research directions. Section 5 concludes this study.

## 2. Methodology

This section introduces the unified benchmark framework, the evaluated model groups, and the task-oriented analysis used in this study.

### 2.1. Overall Framework

To systematically evaluate the applicability, robustness, and deployment potential of YOLO-based models for remote sensing building instance segmentation, this study constructs a unified multidimensional benchmark framework. The framework is designed to compare representative models under consistent data processing, training, inference, and evaluation settings, so that the observed performance differences can be mainly attributed to model characteristics rather than inconsistent experimental protocols.

The benchmark consists of two evaluation groups. The first group is the primary instance segmentation benchmark, which includes models with explicit mask prediction heads: YOLOv8-seg, YOLOv11-seg, YOLO26-seg, and Mask R-CNN. These models are evaluated using both bounding-box-level and mask-level metrics, including detection accuracy, mask quality, inference efficiency, model complexity, convergence behavior, and qualitative visualization. The second group includes RT-DETR-l and RT-DETR-x as auxiliary detection-only Transformer baselines. Because the implemented RT-DETR models do not provide instance mask outputs, they are used only for bounding-box-level detection analysis and are excluded from mask-level segmentation comparisons.

The overall workflow contains four main stages: data preparation, unified training, quantitative evaluation, and visual verification. First, the remote sensing images and annotations are standardized, and semantic building masks are converted into instance-level annotations to make them suitable for instance segmentation models. Second, all evaluated models are trained under the same input resolution, optimizer settings, learning-rate schedule, training epochs, and data augmentation strategy. Third, the trained models are evaluated from multiple perspectives, including detection performance, segmentation accuracy, inference speed, parameter scale, computational cost, and training stability. Finally, convergence curves, confusion matrices, Precision–Recall curves, and representative visual results are used to further interpret the quantitative results and analyze model behavior in complex remote sensing scenes.

### 2.2. Evaluated Models

#### 2.2.1. YOLO-Based Instance Segmentation Models

YOLO-based segmentation models are selected as the main evaluation targets because they provide an end-to-end single-stage solution for object localization and instance mask prediction. Compared with two-stage frameworks, YOLO-seg models generally have lower inference latency and are more suitable for large-scale remote sensing image interpretation and near-real-time deployment. This is particularly important for building extraction tasks, where practical applications often require both high segmentation accuracy and efficient processing of large image volumes [18,19,20,21,22].

This study evaluates three representative YOLO-based segmentation families: YOLOv8-seg, YOLOv11-seg, and YOLO26-seg. YOLOv8-seg is included as a widely used and stable YOLO segmentation baseline. YOLOv11-seg is evaluated because it represents a more recent YOLO-based segmentation framework with favorable accuracy–efficiency characteristics. YOLO26-seg is further included to examine whether newer YOLO-based designs can provide additional benefits for remote sensing building instance segmentation. For each YOLO family, models of different scales are evaluated to analyze the influence of model capacity on detection accuracy, mask quality, inference speed, and computational complexity.

**Figure 1:**
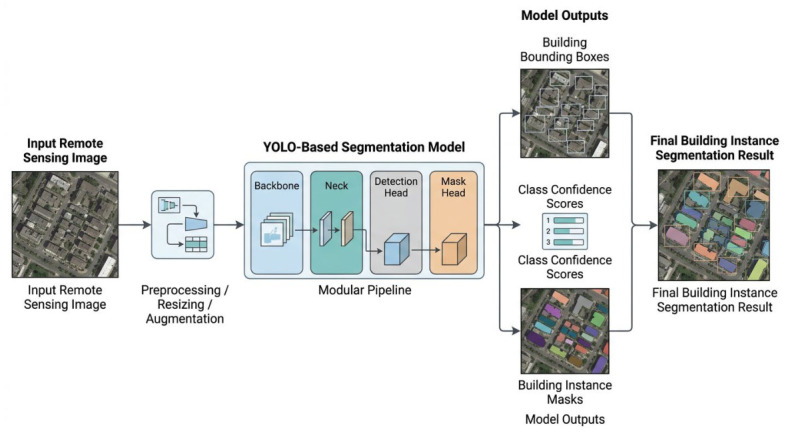
General input–output workflow of the evaluated YOLO-based instance segmentation models.

In the evaluated YOLO-seg models, the input is a remote sensing image, and the outputs include building bounding boxes, confidence scores, and instance-level building masks. The detection branch provides object localization information, while the segmentation branch generates instance masks based on shared multi-scale features. This joint detection–segmentation design is suitable for remote sensing building extraction because the task requires both accurate object localization and fine-grained boundary recovery. The general input–output workflow of the evaluated YOLO-based instance segmentation models is illustrated in Figure 1. In this benchmark, the YOLO-based models are not treated as newly proposed architectures, but as representative single-stage segmentation baselines for systematic comparison under unified experimental settings.

#### 2.2.2. Mask R-CNN

Mask R-CNN is selected as the representative two-stage instance segmentation baseline [3]. Unlike YOLO-based single-stage models, Mask R-CNN first generates candidate regions and then performs classification, bounding-box regression, and mask prediction for each region of interest. This design has been widely used in instance segmentation tasks and provides a meaningful reference for evaluating the advantages and limitations of single-stage YOLO-based models.

**Figure 2:**
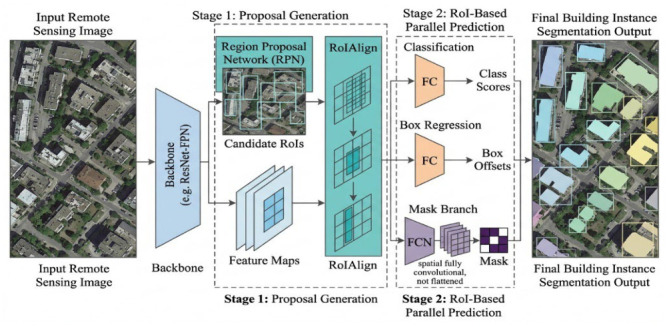
Overall architecture of Mask R-CNN.

In this study, Mask R-CNN is included mainly for cross-paradigm comparison. Its region proposal and RoI-based mask prediction mechanism can provide strong instance-level representation, especially for object localization and mask refinement. However, the two-stage inference process usually introduces higher computational cost and lower throughput, which may limit its efficiency in large-scale remote sensing applications. Therefore, Mask R-CNN serves as a classical two-stage baseline for comparing segmentation accuracy, inference efficiency, and model complexity with YOLO-based segmentation models. Its overall architecture is shown in Figure 2.

#### 2.2.3. RT-DETR

RT-DETR-l and RT-DETR-x are included as auxiliary Transformer-based detection baselines [23]. Transformer-based detectors introduce global context modeling and set prediction mechanisms, which are potentially useful for remote sensing scenes with wide spatial coverage and complex contextual relationships. Including RT-DETR allows this study to compare convolution-dominated YOLO-based detectors with Transformer-based detection models under the same remote sensing building localization setting.

**Figure 3:**
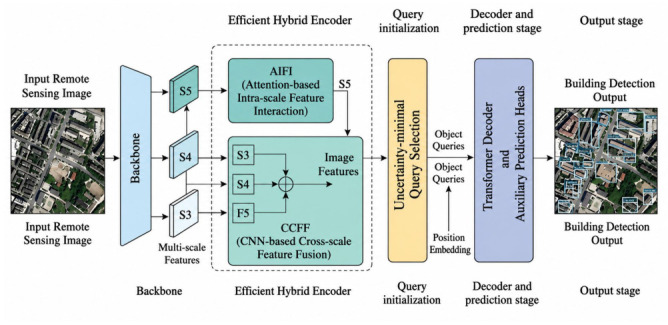
Overall architecture of RT-DETR.

However, the implemented RT-DETR models used in this study do not contain a dedicated instance mask prediction head. Therefore, they are not regarded as instance segmentation models in the primary benchmark. Their evaluation is restricted to bounding-box-level metrics, and they are excluded from mask-level metrics such as P(M), R(M), mAP(M), and mIoU. This setting avoids unfair comparison between mask-predicting segmentation models and detection-only models, while still providing useful reference evidence on the detection-stage behavior of Transformer-based methods. Its overall architecture is shown in Figure 3.

### 2.3. Task Formulation and Model-Suitability Analysis

Remote sensing building instance segmentation requires the model to simultaneously identify building locations and recover instance-level masks. Given an input remote sensing image i, the objective is to predict a set of building instances:

(1) $$Y={\left(b_{i},c_{i},m_{i}\right)}_{i=1}^{N}$$

where $b_{i}$ denotes the bounding box of the $i_{th}$ building instance, $c_{i}$ denotes the class label, $m_{i}$ denotes the corresponding instance mask, and N is the number of detected building instances. Compared with semantic segmentation, this task requires not only pixel-level building extraction but also instance-level separation of adjacent buildings. Compared with object detection, it further requires accurate contour recovery and mask prediction.

This task is particularly challenging in high-resolution remote sensing imagery because buildings often vary greatly in scale, shape, density, and spatial arrangement. Dense building clusters may cause instance adhesion, small buildings may be missed, and shadows, vegetation, roads, and background textures may interfere with boundary localization. Therefore, an effective model should have three types of capabilities: multi-scale feature representation for buildings of different sizes, fine-grained boundary modeling for accurate mask recovery, and efficient inference for large-scale image processing.

From this perspective, YOLO-based segmentation models are expected to be suitable for practical remote sensing building instance segmentation because they combine single-stage inference with joint detection and mask prediction. Their multi-scale feature fusion structure helps detect buildings of different sizes, while their end-to-end prediction mechanism supports efficient deployment. Large-scale YOLO models may provide stronger feature representation and boundary recovery, whereas medium-scale models may offer a better balance among accuracy, speed, and complexity [24].

Mask R-CNN provides a different two-stage segmentation paradigm. Its candidate-region-based design can support instance-level mask refinement, but the additional region proposal and RoI processing steps increase inference cost. Therefore, it is useful as a baseline for analyzing whether the higher computational cost of two-stage segmentation can bring corresponding accuracy advantages in remote sensing building extraction.

RT-DETR represents a Transformer-based detection paradigm. Its global context modeling ability may be useful for building localization in complex scenes, but without a mask prediction head it cannot directly address the complete instance segmentation task. For this reason, RT-DETR is used only to evaluate bounding-box-level detection performance. This distinction is important for ensuring that the benchmark remains methodologically fair and that segmentation conclusions are drawn only from models capable of producing instance masks.

Overall, the methodology of this study is designed to provide a fair and compact comparison of different model paradigms rather than to introduce a new network structure. By evaluating YOLO-based segmentation models, Mask R-CNN, and detection-only RT-DETR baselines under the same data, training, and evaluation settings, this benchmark aims to clarify their respective strengths and limitations in remote sensing building instance segmentation.

## 3. Experiments and Evaluation

### 3.1. Experimental Setup

To ensure fairness and reproducibility in the comparison between different models, this paper trains and evaluates each model under a unified experimental environment. The experimental platform consists of an NVIDIA RTX 4090 GPU with 24 GB VRAM (NVIDIA Corporation, Santa Clara, CA, USA), an Intel i9-12900K CPU (Intel Corporation, Santa Clara, CA, USA), the Ubuntu 20.04 operating system (Canonical Ltd., London, UK), the PyTorch 2.6.0 deep learning framework (Meta Platforms, Inc., Menlo Park, CA, USA), and CUDA version 12.4 (NVIDIA Corporation, Santa Clara, CA, USA). All input images were uniformly resized to 512 × 512, with a batch size of 24 and 120 training epochs. The AdamW optimizer was used with an initial learning rate of 0.001, momentum of 0.937, and weight decay of 0.0005, along with a cosine-smoothed learning rate schedule.

To improve the statistical reliability of the benchmark comparison, each evaluated model was trained independently three times using different random seeds. For all runs, the same WHU data split, input resolution, optimizer, learning-rate schedule, training epochs, and data augmentation strategy were maintained to ensure a fair comparison. The reported results are based on the three independent random-seed runs, and the corresponding statistical reliability analysis is presented as the mean and standard deviation of the main evaluation metrics.

The evaluated models were divided into two groups. The first group consisted of mask-predicting instance segmentation models, including YOLOv8-seg, YOLOv11-seg, YOLO26-seg, and Mask R-CNN. These models were used in the primary instance segmentation benchmark. The second group consisted of RT-DETR-l and RT-DETR-x, which were used only as auxiliary detection-only baselines for bounding-box-level comparison.

### 3.2. Dataset

This study utilized the WHU Building Extraction Dataset [25]. The WHU dataset comprises 8188 non-overlapping RGB images, each with a size of 512 × 512 pixels. The images were captured in Christchurch, New Zealand, with a spatial resolution of 0.3 m. According to the official guidelines, a total of 4735 images were allocated to the training set, 1036 to the validation set, and 2416 to the test set.

To comprehensively evaluate the robustness and generalization capabilities of the proposed model when faced with geographical variations, sensor noise, and complex urban structures, the Inria Aerial Image Labeling dataset was introduced for cross-domain benchmarking and controlled degradation analysis [26]. Unlike the source domain, the Inria dataset features dense high-rise buildings in five major cities across Europe and the United States, representing more challenging urban mapping scenarios.

#### 3.2.1. WHU Dataset

The original WHU Building Dataset provides semantic building masks. To adapt the dataset to instance segmentation models, the semantic masks were converted into instance-level annotations using a connected-component-based procedure implemented with OpenCV. First, each semantic mask was binarized, where building pixels were treated as foreground and all other pixels as background. Second, 8-connected component analysis was applied using cv2.connectedComponentsWithStats, and each connected foreground region was initially regarded as one building instance. For each connected component, the bounding box, pixel area, and external contour were extracted. The contour was then converted into a polygon annotation using cv2.findContours, and polygon simplification was performed using the Douglas–Peucker algorithm implemented by cv2.approxPolyDP. Components with invalid contours, fewer than three polygon vertices, or extremely small areas were removed to avoid generating invalid annotations. The official training, validation, and test split of the WHU dataset was strictly preserved throughout the conversion process.

Because adjacent buildings may be incorrectly merged when their semantic masks are spatially connected, and fragmented roof regions may occasionally be over-separated, we performed both automatic sanity checks and manual quality inspection on the generated instance annotations. The automatic checks included verifying whether each instance polygon was valid, whether the bounding box was within the 512 × 512 image boundary, whether the polygon area was consistent with the corresponding connected-component area, and whether the number of generated instances matched the number of valid connected components in each mask. In addition, a stratified manual inspection was conducted on 300 randomly selected images from the training, validation, and test sets, covering sparse buildings, dense building clusters, shadow-affected scenes, and cases with adjacent buildings. The inspection mainly focused on potential annotation errors, including merged adjacent buildings, over-separated building fragments, boundary simplification errors, and invalid polygons.

**Figure 4:**
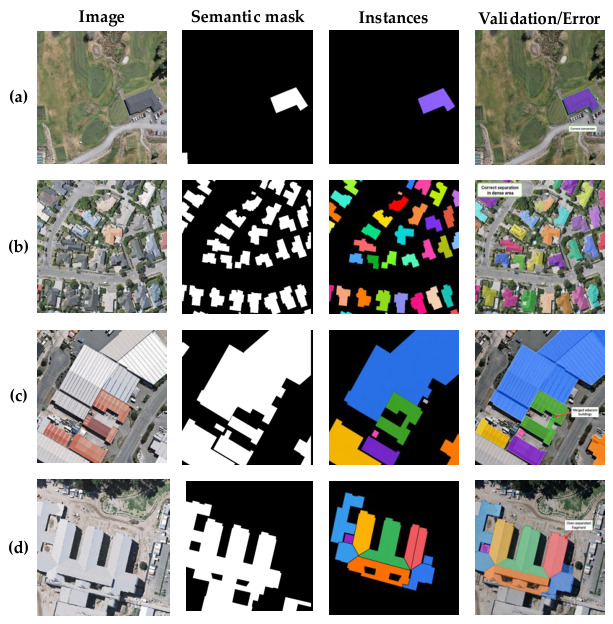
Examples of semantic-to-instance annotation conversion and validation. From left to right, each row shows the RGB image, original semantic mask, generated instance annotation, and validation overlay. The examples include (**a**) correct conversion for isolated buildings; (**b**) correct separation in dense building areas; (**c**) merged adjacent buildings; and (**d**) over-separated building fragments.

**Figure 5:**
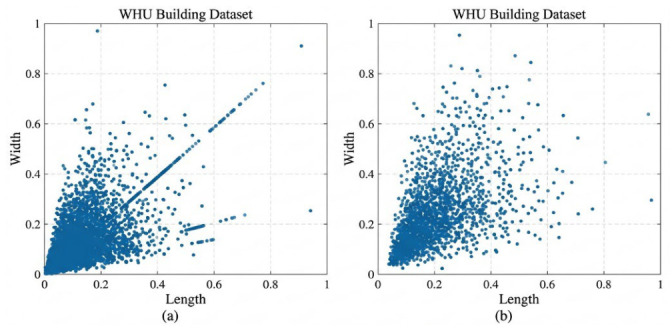
Scale statistics of building objects in the WHU dataset. (**a**) Distribution of building sizes in the full dataset; (**b**) distribution of small buildings.

Table 1 showed that most converted annotations were consistent with the original semantic masks. The main ambiguous cases occurred when neighboring buildings were directly connected in the semantic masks or when narrow gaps between roofs were not visible. In such cases, connected component analysis may treat multiple adjacent buildings as a single instance. Since this limitation originates from the semantic-mask representation rather than the conversion algorithm itself, these cases were explicitly reported as annotation ambiguity. Representative correct and ambiguous conversion examples are shown in Figure 4. Figure 5 presents the scale statistics of building objects within the WHU dataset.

#### 3.2.2. Inria Dataset

For the Inria experiments, all models were trained and tested independently on the Inria dataset using the same preprocessing pipeline, input size, optimizer, training schedule, data augmentation strategy, and evaluation metrics as those used for WHU. The semantic building masks were converted into instance-level annotations using the same connected-component-based procedure described above.

### 3.3. Evaluation Metrics

To objectively evaluate the performance of different models in the building instance segmentation task on remote sensing imagery, this paper constructs an evaluation framework based on three dimensions: detection accuracy, segmentation quality, and training stability. Compared to natural scenes, remote sensing building targets typically exhibit characteristics such as wide-ranging scales, high instance density, and regular boundaries that are prone to interference from shadows and complex backgrounds. Therefore, evaluation metrics must not only reflect whether targets are correctly detected but also characterize the model’s ability to recover building contours, boundary details, and the separation of adjacent instances. Based on this, this paper conducts statistical analysis at two levels: bounding box detection results and mask segmentation results, using Precision, Recall, mAP@0.5, mIOU, mAP@0.5:0.95, and F1-score as core metrics.

For instance segmentation tasks, the Intersection-over-Union is a key metric for measuring the degree of overlap between the predicted mask and the ground-truth mask, defined as

(2) $$IoU=\frac{\left|M_{p}\cap M_{g}\right|}{\left|M_{p}\cup M_{g}\right|},$$

where $M_{p}$ and $M_{g}$ represent the predicted mask and the ground-truth mask, respectively. The average Intersection-Over-Union (IOU) mIoU is obtained by averaging over all samples in the test set:

(3) $$mIoU=\frac{1}{N}\sum_{i=1}^{N}IoU_{i}$$

where N is the number of samples. This metric directly reflects the model’s ability to recover the main regions and boundary shapes of buildings, and is of great significance for evaluating the contour integrity and separation of adjacent instances in densely built-up areas.

Regarding the evaluation of detection and segmentation accuracy, this paper further employs precision and recall:

(4) $$\;\mathrm{Precision}\;=\frac{TP}{TP+FP},\;\;\mathrm{Recall}\;=\frac{TP}{TP+FN},$$

where TP, FP, and FN represent true positives, false positives, and false negatives, respectively. Precision is used to measure the model’s ability to control false positives, while Recall is used to measure its ability to control false negatives. For remote sensing building instance segmentation, a low Precision typically indicates that the model tends to misclassify roads, shadows, or background textures as buildings, whereas a low Recall suggests that the model struggles to identify small-scale buildings, occluded buildings, or targets in densely populated areas. To comprehensively describe the model’s overall performance across different thresholds, this paper employs the average precision AP and its mean mAP:

(5) $$AP=\int_{0}^{1}P(R)dR,\;mAP=\frac{1}{C}\sum_{k=1}^{C}AP_{k}$$

where C is the number of classes? Additionally, we calculate mAP@0.5(B) at the bounding box level, as well as mAP@0.5(M) at the mask level. Among these, mAP@0.5 the primary one reflects the model’s overall detection capability, while mAP@0.5:0.95 placing higher demands on localization and boundary accuracy, making it more suitable for evaluating contour recovery and fine-grained separation capabilities in remote sensing building segmentation.

Furthermore, to analyze the model’s ability to balance Precision and Recall, this paper introduces F1-score:

(6) $$F1=2\cdot \frac{\;\mathrm{Precision}\;\cdot \;\mathrm{Recall}\;}{\;\mathrm{Precision}\;+\;\mathrm{Recall}\;}.$$

F1-score This metric comprehensively reflects the trade-off between suppressing false positives and reducing false negatives, making it suitable for evaluating threshold stability in practical applications. For remote sensing scenarios, a high and stable F1-score value indicates that the model can maintain a high building detection rate while effectively reducing misclassifications caused by complex backgrounds.

Regarding evaluation during the training process, this paper also analyzes the model’s convergence through the loss function. The total loss can be expressed as

(7) $$L=\lambda_{cls}L_{cls}+\lambda_{box}L_{box}+\lambda_{seg}L_{seg}+\lambda_{dfl}L_{dfl},$$

where $L_{cls}$, $L_{box}$, $L_{seg}$, and $L_{dfl}$ represent the classification loss, bounding box regression loss, segmentation loss, and distribution focus loss, respectively, and λ is the corresponding weight coefficient. By tracking changes in these loss components and validation set metrics, the model’s training stability and generalization ability can be further assessed.

In summary, the evaluation metric system adopted in this paper comprehensively characterizes the model’s actual performance in the remote sensing building instance segmentation task from multiple dimensions, including region overlap, detection capability, boundary accuracy, threshold robustness, and training stability. Among these, mIoU we mAP@0.5:0.95(M) place greater emphasis on segmentation quality and boundary recovery capability, while Precision, Recall, and F1-score are used to characterize the balance between false positives and false negatives. Changes in the loss function provide a basis for analyzing the model optimization process. This establishes a unified and rigorous evaluation standard for comparing experimental results and analyzing the optimal model.

### 3.4. Implementation Details

The implementation of the method described in this paper follows a standardized workflow within a unified experimental framework, divided into three key stages: data preparation and preprocessing, model training and optimization, and inference and result generation. The goal is to achieve fine-grained instance segmentation of building targets in remote sensing imagery. First, during the data preparation stage, high-resolution remote sensing imagery and its corresponding annotations undergo standardized processing, including image resizing, annotation format conversion, and data augmentation operations. Through strategies such as random scaling, cropping, flipping, and mosaic augmentation, the model’s adaptability to scale variations, complex backgrounds, and dense object distributions is effectively enhanced, thereby constructing a training dataset with good generalization capabilities. At the same time, data partitioning and input resolution settings are standardized to ensure fair comparison of different models under identical experimental conditions.

During the model training phase, this paper employs a unified training strategy to perform end-to-end optimization on YOLO-series instance segmentation models and comparison models. The training process is based on input images, utilizing a feature extraction network to obtain multi-scale semantic information, and simultaneously outputs object bounding boxes and pixel-level masks through the collaborative action of the detection and segmentation branches. Model optimization employs a multi-task joint loss function to comprehensively constrain classification, localization, and segmentation errors, thereby achieving a synergistic improvement in detection and segmentation performance. During training, the model’s convergence and generalization capabilities are evaluated by continuously monitoring trends in training loss and validation set metrics (such as Precision, Recall, and mAP). Additionally, a unified optimizer and learning rate scheduling strategy are employed to ensure stable convergence across different models under consistent training conditions, thereby guaranteeing the reliability and comparability of experimental results.

During the inference stage, an efficient end-to-end prediction workflow is established. For input remote sensing imagery, the model first performs object detection to generate candidate building regions and their class information, followed by the segmentation branch generating corresponding mask results for each instance. For YOLO-series models, mask generation is based on a mechanism that fuses prototype masks with coefficients, significantly reducing computational overhead while ensuring segmentation accuracy. The generated low-resolution masks are further upsampled to the original image scale to obtain high-resolution segmentation results consistent with the real-world scene. For Mask R-CNN, instance masks are generated through candidate region refinement and the mask prediction branch. In contrast, RT-DETR-l and RT-DETR-x were evaluated only as detection baselines in this study because the implemented models do not provide instance mask outputs. Therefore, RT-DETR was included only in bounding-box-level comparisons and excluded from mask-level metric comparisons.

### 3.5. Results and Analysis

#### 3.5.1. Accuracy Comparison Analysis

Table 2 summarizes the detection and instance segmentation performance of all evaluated models on the WHU test set. Precision, Recall, mAP@0.5, and mAP@0.5:0.95 were calculated at both the bounding-box level (B) and mask level (M). Since RT-DETR-l and RT-DETR-x do not output instance masks in the implemented setting, they were evaluated only using bounding-box-level metrics and were excluded from mask-level comparisons.

Overall, the YOLO-based segmentation models achieved competitive performance under the unified training and evaluation protocol. Among the mask-predicting models, high-capacity YOLO-seg variants generally obtained stronger detection and segmentation results than lightweight variants, indicating that increased model capacity improves feature representation and boundary recovery. However, the improvement was not strictly linear, especially among medium- and large-scale models, where the performance gaps became relatively small.

YOLOv11x-seg showed the strongest overall mask-level performance in the current WHU setting, achieving the highest mAP@0.5(M), mAP@0.5:0.95(M), and mIoU among the comparable segmentation models. This suggests that it has favorable ability to recover building masks and maintain boundary consistency under strict IoU thresholds. YOLO26x-seg achieved the highest mAP@0.5(B) and Recall(B), showing strong building target coverage and competitive detection performance. Its mask-level results were also close to those of YOLOv11x-seg, indicating that both models have a high performance ceiling for remote sensing building instance segmentation.

Compared with YOLO-based models, Mask R-CNN achieved reasonable precision but lower recall and lower high-threshold mAP values. This indicates that the two-stage baseline can suppress false positives to some extent, but may miss more building instances in dense or complex scenes. RT-DETR-l and RT-DETR-x provided only detection-level results. Under the current setting, their bounding-box performance did not exceed the best YOLO-seg models, and they cannot be directly compared in terms of mask quality.

**Figure 6:**
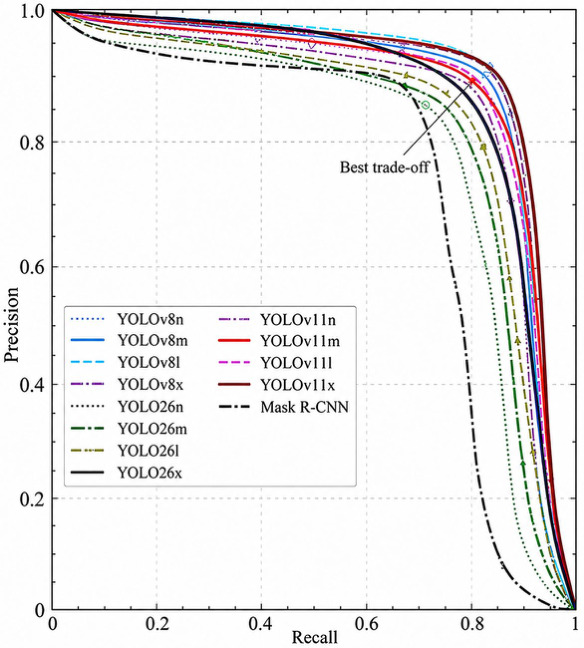
Comparison of precision–recall curves for different segmentation models.

The Precision–Recall curves in Figure 6 further support the trends observed in Table 2. The curves of high-capacity YOLO-seg models are generally closer to the upper-right region, indicating a better balance between precision and recall. In contrast, lightweight models show a clearer performance decline when recall increases, suggesting that they are more sensitive to false positives and missed detections in complex remote sensing scenes.

**Figure 7:**
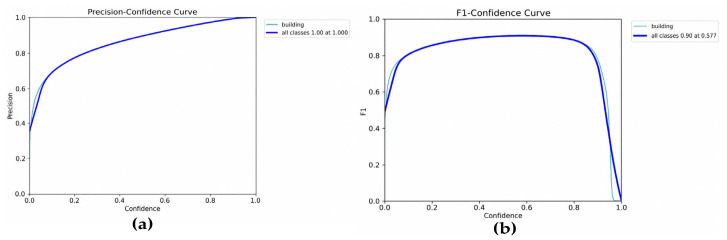
Precision–confidence and F1–confidence curves for the YOLOv11x-seg model. Precision–confidence curve (**a**); F1–confidence curve (**b**).

Figure 7 presents the Precision–Confidence and F1–Confidence curves of YOLOv11x-seg. The results indicate that YOLOv11x-seg maintains reliable predictions over a broad confidence range and achieves a stable balance between precision and recall at moderate confidence thresholds. This threshold stability is useful for practical remote sensing applications, where the balance between missed buildings and false alarms may vary according to operational requirements. 

To evaluate the statistical reliability of the main high-accuracy comparison, Table 3 reports the results of three independent random-seed runs for YOLOv8x-seg, YOLOv11x-seg, and YOLO26x-seg. The repeated-seed results show that the relative ranking among these representative high-capacity models is generally stable. YOLOv11x-seg maintains a slight and consistent advantage in high-threshold mask accuracy, while YOLO26x-seg remains highly competitive in detection and mask prediction. Because the numerical differences between the best-performing models are small, these results should be interpreted as benchmark-specific trends rather than evidence of universal model superiority.

In summary, the WHU accuracy comparison shows that YOLO-based segmentation models are effective for remote sensing building instance segmentation. YOLOv11x-seg is the strongest accuracy-oriented model in this benchmark, whereas YOLO26x-seg provides highly competitive detection coverage and mask performance. The following sections further analyze inference efficiency, model complexity, convergence behavior, and robustness to support application-oriented model selection.

#### 3.5.2. Efficiency and Complexity Analysis

In addition to segmentation accuracy, inference efficiency and model complexity are important factors for practical remote sensing building extraction, especially when large image volumes or near-real-time processing are required. Table 4 summarizes the inference time, FPS, parameter size, and GFLOPs of all evaluated models under the same hardware and input-resolution settings.

The results show a clear trade-off among accuracy, speed, and computational cost. Lightweight YOLO-seg models achieve the highest throughput and have the lowest parameter and FLOP requirements, making them suitable for resource-constrained or real-time scenarios. However, their mask-level accuracy is generally lower than that of medium- and large-scale models. In contrast, x-scale models such as YOLOv11x-seg and YOLO26x-seg provide stronger accuracy but require higher computational cost and show reduced FPS. Therefore, these models are more suitable for accuracy-oriented mapping tasks where inference latency is less restrictive.

Among the evaluated models, YOLOv11m-seg provides the most favorable overall balance. It achieves competitive detection and mask accuracy while maintaining 85.0 FPS with 22.421 M parameters and 72.866 GFLOPs, which is substantially more efficient than x-scale models. YOLOv11x-seg remains the preferred choice when the highest mask accuracy is required, whereas YOLO26x-seg provides strong detection coverage with a similar high-complexity deployment cost.

Compared with YOLO-seg models, Mask R-CNN and RT-DETR show lower inference efficiency under the current setting. Mask R-CNN is affected by region proposal and RoI-based processing, while RT-DETR introduces additional computational overhead from Transformer-based feature interaction. Although these models are useful as representative baselines, their speed and deployment efficiency are less favorable for large-scale remote sensing building instance segmentation in this benchmark.

**Figure 8:**
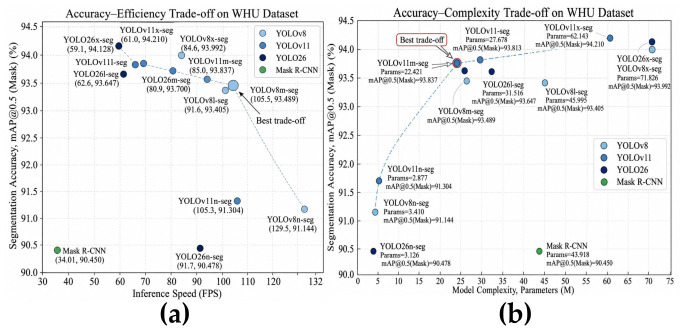
Efficiency-accuracy comparison of segmentation models (**a**); comparison of complexity and accuracy for segmentation models (**b**).

Figure 8 further illustrates the accuracy–efficiency and accuracy–complexity relationships. Overall, lightweight models are located in the high-speed but moderate-accuracy region, x-scale models are located in the high-accuracy but high-cost region, and YOLOv11m-seg lies close to the practical Pareto front. Therefore, YOLOv11m-seg can be regarded as the most balanced deployment-oriented model, while YOLOv11x-seg is more appropriate for accuracy-first applications.

#### 3.5.3. Visualization and Convergence Analysis

To complement the quantitative comparisons, this section briefly analyzes the training stability and representative visual results of the evaluated segmentation models. Since the numerical accuracy, efficiency, and complexity results have already been reported in Table 2, Table 3 and Table 4, the following analysis focuses only on the main qualitative and convergence-related observations.

Table 5 reports the late-stage fluctuations of three representative high-capacity YOLO-seg models over the final 20 recorded epochs. All three models show stable convergence in the later training stage, with small standard deviations in the main mask-level validation metrics. Among them, YOLOv11x-seg exhibits relatively low fluctuations in Precision(M), Recall(M), mAP@0.5(M), and mAP@0.5:0.95(M), suggesting that its final performance is not caused by a temporary metric peak but is supported by stable late-stage optimization. However, its validation loss terms are not consistently the lowest, indicating that convergence stability should be interpreted together with final mask accuracy rather than from loss values alone.

**Figure 9:**
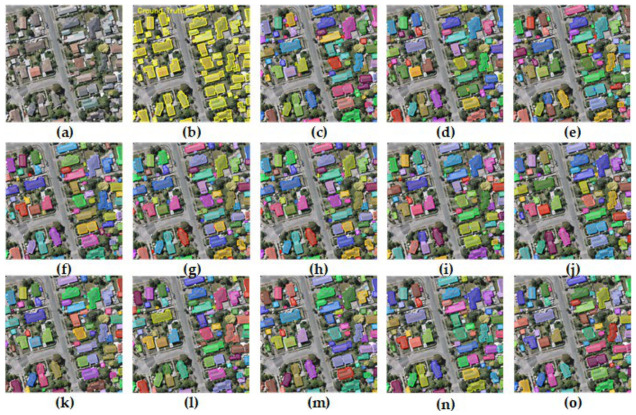
Comparison of detection and segmentation results from different models in typical remote sensing scenarios. Experimental results on the WHU dataset. Methods are listed from left to right and top to bottom as follows: (**a**) image; (**b**) Ground Truth (GT); (**c**) YOLOv8n-seg; (**d**) YOLOv8m-seg; (**e**) YOLOv8l-seg; (**f**) YOLOv8x-seg; (**g**) YOLOv11n-seg; (**h**) YOLOv11m-seg; (**i**) YOLOv11l-seg; (**j**) YOLOv11x-seg; (**k**) YOLO26n-seg; (**l**) YOLO26m-seg; (**m**) YOLO26l-seg; (**n**) YOLO26x-seg; (**o**) Mask R-CNN.

Figure 9 presents representative detection and segmentation results on WHU test images. Overall, high-capacity YOLO-seg models produce more complete building masks and clearer boundaries than lightweight variants, especially in dense building areas and scenes with shadow or background interference. YOLOv11m-seg and YOLOv11x-seg show relatively good instance separation and boundary consistency, while lightweight models are more likely to show local omissions or fragmented masks. Mask R-CNN provides reasonable instance predictions but shows lower completeness in some dense scenes, which is consistent with its lower recall in Table 2.

**Figure 10:**
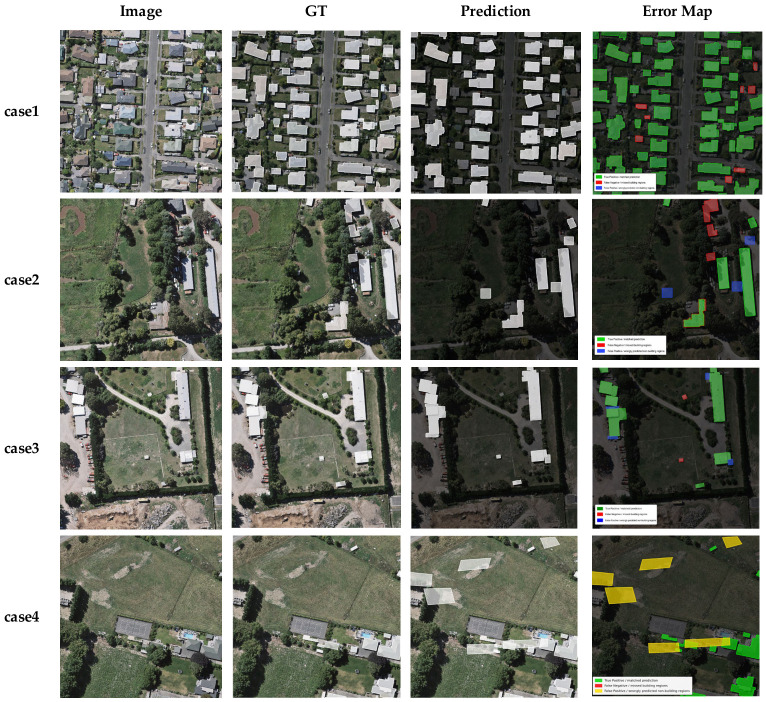
Typical false-negative and false-positive cases of YOLOv11x-seg on the WHU test set. Green, red, and yellow or blue indicate true-positive regions, false-negative regions, and false-positive regions, respectively. case1: small buildings; case2: buildings partially affected by shadows or vegetation occlusion; case3: adjacent roofs with narrow gaps; case4: buildings with weak roof textures.

To further analyze the remaining limitations of the evaluated models, Figure 10 presents representative false-negative and false-positive cases of YOLOv11x-seg on the WHU test set. The error maps are used to distinguish correctly matched building regions, missed building regions, and wrongly predicted non-building regions. 

The false-negative cases mainly occur for small buildings, buildings with weak roof textures, and buildings partially affected by shadows or vegetation occlusion. In these cases, the model may produce incomplete masks or miss the building instances entirely because the roof appearance is visually similar to the surrounding background. Another typical failure mode is observed in dense building areas, where adjacent roofs with narrow gaps may lead to incomplete instance separation or locally fragmented masks. False-positive cases mainly occur on building-like background objects, including bright impervious surfaces, road fragments, parking areas, and rectangular non-building structures. These regions may share similar spectral intensity, regular geometry, or high-contrast boundaries with building roofs, causing the model to incorrectly classify them as buildings.

These failure cases indicate that, although YOLOv11x-seg achieves strong overall performance in the quantitative benchmark, fine-grained discrimination between buildings and visually similar background objects remains challenging. Small-object sensitivity, boundary completeness, and robustness to shadow, vegetation occlusion, and bright non-building surfaces are still important directions for future improvement.

Overall, the visualization and convergence results are consistent with the quantitative findings. YOLOv11x-seg is more suitable for accuracy-oriented applications, whereas YOLOv11m-seg provides a more practical balance between accuracy, speed, and model complexity. These observations further support the model-selection conclusions drawn from the accuracy and efficiency analyses. Summary of representative models is shown in Table 6.

### 3.6. Cross-Dataset Transferability and Controlled-Degradation Robustness Analysis

To strengthen the controlled-degradation robustness analysis beyond training curves, confusion matrices, and qualitative visualization, we conducted three complementary evaluations. First, we performed zero-shot WHU-to-Inria testing in which the model trained on WHU was directly evaluated on the Inria validation set without target-domain fine-tuning. Second, we conducted independent in-domain training and testing on the Inria dataset using different initialization strategies, including WHU-Init and COCO-Init. Third, we performed controlled degradation tests under shadow/occlusion and Gaussian blur conditions without additional fine-tuning. These experiments were designed to assess cross-dataset transferability, target-domain reproducibility, initialization sensitivity, and degradation-specific robustness.

#### 3.6.1. Zero-Shot Cross-Dataset Transferability Challenge

**Figure 11:**
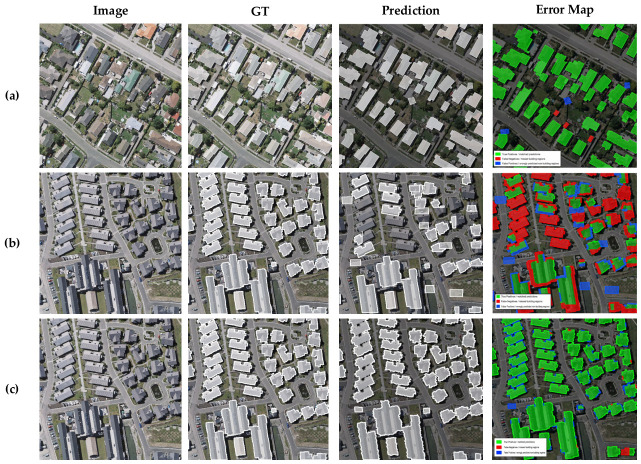
Visual comparison of WHU in-domain testing, zero-shot WHU-to-Inria testing, and Inria in-domain testing. (**a**) WHU in-domain; (**b**) Zero-shot WHU → Inria; (**c**) Inria in-domain. True Positive: white; False Positive: red; False Negative: blue.

In preliminary zero-shot cross-dataset testing, a model trained on WHU was directly evaluated on the Inria validation set without target-domain fine-tuning. The mask mAP@0.5 decreased markedly to approximately 0.230, which is far lower than its corresponding performance on the WHU test set. This sharp degradation indicates a clear domain-shift effect between the two datasets. The WHU and Inria datasets exhibit differences in building layout, roof appearance, spatial density, shadow distribution, and background texture. As a result, features learned from a single source region may not be directly transferable to another geographic domain. This finding suggests that high performance on one remote-sensing benchmark should not be interpreted as evidence of direct global applicability, and that target-domain adaptation, retraining, or carefully designed transfer-learning strategies remain necessary for large-scale operational mapping. Figure 11 shows Visual comparison of WHU in-domain testing, zero-shot WHU-to-Inria testing, and Inria in-domain testing, respectively.

Compared with the WHU in-domain results, the zero-shot WHU-to-Inria predictions show a clear increase in missed buildings, fragmented masks, boundary errors, and false-positive regions. These visual errors are consistent with the sharp decrease in mask mAP@0.5 observed in the zero-shot experiment.

The degradation can be mainly attributed to domain shift between the WHU and Inria datasets. The two datasets differ in geographic region, building layout, roof appearance, object density, illumination condition, shadow distribution, and background texture. Therefore, features learned from the WHU source domain cannot be directly transferred to the Inria target domain without performance loss. After target-domain training on Inria, the predicted masks become more complete, building boundaries are better recovered, and the number of false detections is reduced. This comparison provides intuitive visual evidence that target-domain adaptation or retraining is necessary for reliable cross-dataset remote sensing building instance segmentation.

#### 3.6.2. Target-Domain Reproducibility on the Inria Dataset

To investigate the performance limits of different YOLO model architectures on the target domain and the impact of different pre-trained weights on remote sensing feature transfer, we designed a systematic in-domain ablation study on the Inria dataset. The experiments conducted an in-depth comparison between YOLOv8x-seg, YOLOv11x-seg, and YOLO26x-seg, and evaluated two weight initialization strategies: 1. WHU-Init: Transfer learning using the best weights trained on the WHU dataset; 2. COCO-Init: Training from scratch using the official standard general-purpose weights.

The results in Table 7 show that the effect of WHU-based initialization is model-dependent. For YOLOv8x-seg, WHU-Init achieves a mask mAP@0.5 of 0.817, slightly higher than the 0.809 obtained with COCO-Init, suggesting that source-domain remote-sensing features can provide a modest positive transfer effect for this architecture. In contrast, YOLOv11x-seg obtains 0.819 with WHU-Init and 0.821 with COCO-Init, while YOLO26x-seg obtains 0.814 with WHU-Init and 0.819 with COCO-Init. These results indicate that, for the newer high-capacity architectures, WHU initialization does not consistently improve performance on Inria and may slightly constrain target-domain optimization. A plausible explanation is that stronger feature extraction and attention-based modules can more effectively reconstruct target-domain representations when initialized from more generic visual priors, whereas source-domain remote-sensing weights may encode dataset-specific appearance patterns, such as roof color, vegetation context, and shadow geometry. However, the numerical gaps are small; therefore, the results should be interpreted as evidence of initialization sensitivity rather than as a definitive favorable performance of one pretraining strategy across all conditions.

Among the evaluated configurations on Inria, YOLOv11x-seg with COCO-Init achieves the highest mask mAP@0.5 of 0.821, together with Precision(M) of 0.851 and Recall(M) of 0.768. YOLO26x-seg with COCO-Init reaches the same Precision(M) level as YOLOv8x-seg with WHU-Init and obtains a competitive mask mAP@0.5 of 0.819. These results are consistent with the WHU benchmark in the sense that high-capacity YOLO-series segmentation models remain the strongest group of models under the second dataset setting. Nevertheless, the relatively small differences among YOLOv8x-seg, YOLOv11x-seg, and YOLO26x-seg indicate that the Inria results mainly support dataset-level reproducibility of the overall trend, rather than a large absolute performance gap among the high-capacity models.

#### 3.6.3. Controlled-Degradation Robustness Evaluation

To further examine the robustness of the representative model under controlled image degradation, additional stress tests were conducted on the validation set without model fine-tuning. Table 8 reports the performance of YOLOv11x-seg under the clean baseline and two degradation conditions: shadow/occlusion and Gaussian blur. The relative drop was calculated using mask mAP@0.5 as the reference metric, with the clean baseline value of 0.8168 serving as the denominator. Under shadow/occlusion, mask mAP@0.5 decreases from 0.8168 to 0.8023, corresponding to a relative drop of 1.78%. Under Gaussian blur, mask mAP@0.5 decreases to 0.7066, corresponding to a relative drop of 13.50%. These values indicate that the model is more tolerant to illumination attenuation and partial occlusion than to severe loss of high-frequency spatial details.

The shadow/occlusion results suggest that YOLOv11x-seg does not rely exclusively on local brightness or roof-color cues for building segmentation. Even when part of the image is affected by irregular dark regions, the model maintains a mask mAP@0.5 of 0.8023, Precision(M) of 0.8372, and Recall(M) of 0.7494. The limited performance drop implies that the model can still exploit geometric structure, contextual continuity, and building-level spatial patterns to recover a large proportion of target instances. This property is important for remote-sensing applications, where cloud shadows, terrain shadows, and building self-shadows frequently reduce local spectral separability.

In contrast, Gaussian blur produces a more substantial degradation. The mask mAP@0.5 decreases from 0.8168 to 0.7066, while Recall(M) decreases from 0.7632 to 0.6550. This indicates that the removal of high-frequency details weakens the model’s ability to detect and delineate building boundaries, especially for small buildings, narrow roof structures, and adjacent instances with weak separation. Nevertheless, Precision(M) remains 0.7821 under the blur condition, suggesting that the model still preserves a relatively conservative prediction behavior and does not generate excessive false positives in heavily degraded regions. Therefore, the model exhibits moderate robustness to optical defocus, but accurate boundary recovery remains dependent on sufficient edge and texture information.

Overall, the cross-dataset and controlled-degradation analyses provide complementary evidence for the applicability of YOLO-based instance segmentation models in remote-sensing building extraction. The Inria experiments show that the relative competitiveness of high-capacity YOLO-seg models can be reproduced under an external dataset after in-domain training, while the zero-shot results confirm that direct cross-domain transfer remains challenging. The degradation experiments further show that YOLOv11x-seg is relatively stable to shadow/occlusion but more sensitive to severe blur, which is consistent with the physical dependence of building boundary extraction on high-frequency image information. Therefore, the results support the practical potential of YOLOv11x-seg and related high-capacity YOLO-seg models for large-scale remote-sensing building instance segmentation, while also indicating that domain adaptation, target-domain retraining, and image-quality control remain important prerequisites for reliable deployment in heterogeneous geographic and imaging conditions.

## 4. Discussion

The results of this study provide several insights into the applicability of YOLO-based instance segmentation models for remote sensing building extraction. Rather than indicating universal superiority of a specific model family, the benchmark suggests that single-stage YOLO-seg frameworks can offer a favorable compromise between mask quality, inference efficiency, and model complexity when they are trained and evaluated under consistent conditions. This is particularly relevant for remote sensing applications, where large image coverage, dense building distributions, and practical deployment constraints often require models that can maintain acceptable segmentation accuracy without excessive computational cost.

The performance differences among the evaluated YOLO-seg models also indicate that model capacity should be interpreted together with the intended application scenario. Larger models generally benefit from stronger feature representation and can improve fine-grained mask prediction, especially in scenes involving dense buildings, irregular roof structures, and complex background textures. However, the advantage of high-capacity models is accompanied by increased computational cost and parameter complexity. Therefore, for accuracy-oriented mapping tasks, larger YOLO-seg variants may be preferred, whereas medium-scale models may be more suitable for real-time interpretation or resource-constrained deployment. This trade-off is important because remote sensing building extraction is not only an accuracy-driven task but also an engineering-oriented problem involving processing speed, memory consumption, and scalability.

The comparison with Mask R-CNN and detection-only RT-DETR further highlights the need to distinguish between detection performance and instance segmentation performance. Mask R-CNN provides a representative two-stage instance segmentation baseline, but its region proposal and RoI-based processing introduce additional computational overhead. RT-DETR, on the other hand, represents a Transformer-based detection paradigm with strong object localization capability, but it was not evaluated as a mask-predicting instance segmentation model in this study because the implemented setting did not include a dedicated mask prediction head. Therefore, its role in this benchmark should be understood as an auxiliary detection-level reference rather than a direct mask-level competitor. This distinction is important for avoiding an asymmetric interpretation of cross-paradigm comparisons.

The cross-dataset experiments show that direct transfer from WHU to Inria remains challenging, whereas target-domain training substantially improves performance on the Inria dataset. This observation suggests that the evaluated models are sensitive to domain differences caused by imaging conditions, geographic styles, building morphology, spatial resolution, and annotation characteristics. In practical applications, this means that a model performing well on one benchmark dataset may not be directly transferable to another region or sensor setting without adaptation. Therefore, target-domain retraining, domain adaptation, or carefully designed data augmentation strategies are necessary when applying such models to heterogeneous remote sensing scenarios.

The controlled degradation experiments provide additional evidence regarding model robustness. The relatively smaller performance decrease under shadow/occlusion suggests that YOLOv11x-seg can still exploit surrounding contextual cues when part of a building is locally obscured. In contrast, Gaussian blur causes a more substantial performance reduction because it weakens high-frequency edge and texture information that is essential for separating adjacent buildings and recovering accurate object boundaries. This result is consistent with the characteristics of building instance segmentation, where precise boundary delineation often depends on roof edges, corners, and local contrast. Therefore, image quality control remains an important prerequisite for reliable building extraction, especially in scenarios involving defocus, low-resolution imagery, motion blur, or compression-induced detail loss.

Several limitations should also be noted. First, although the benchmark includes WHU and Inria datasets as well as additional robustness tests, the conclusions remain dataset-specific and setting-specific. More datasets, cross-regional experiments, and multi-sensor evaluations are required before broader generalization claims can be made. Second, the controlled degradation analysis currently focuses only on shadow/occlusion and Gaussian blur. Other degradation factors, such as random noise, atmospheric interference, compression artifacts, seasonal variation, illumination differences, and scale changes, should be systematically examined in future work. Third, this study mainly compares models under unified training settings, but does not fully explore the sensitivity of each model to hyperparameters, data augmentation strategies, training schedules, or input resolution. These factors may influence the relative ranking of different models, especially for Transformer-based methods and high-capacity segmentation networks.

In addition, the instance annotations used in this study were generated from semantic building masks using a connected-component-based conversion strategy. Although this provides a reproducible way to construct instance-level labels, it may introduce annotation uncertainty in densely built-up areas. Adjacent buildings that are connected in the semantic mask may be treated as a single instance, whereas fragmented roof regions may occasionally be separated into multiple instances. Therefore, the generated annotations should be regarded as instance-level approximations rather than fully manual instance annotations. Future work could improve annotation quality through manual refinement, boundary-aware post-processing, or instance separation strategies designed specifically for dense urban scenes.

Future research can extend this benchmark in several directions. First, more remote sensing building datasets, cross-city test areas, and multi-source imagery should be included to further evaluate cross-domain generalization. Second, lightweight and boundary-aware segmentation architectures should be investigated to improve the balance between segmentation accuracy and deployment efficiency. Third, multimodal remote sensing data, such as multispectral, SAR, and LiDAR data, could be incorporated to enhance building discrimination under occlusion, weak texture, and complex background conditions. Finally, with the rapid development of foundation models and general-purpose vision models, future benchmarks should also evaluate their adaptability to remote sensing building instance segmentation under unified and reproducible experimental protocols.

## 5. Conclusions

This study established a unified multidimensional benchmark for remote sensing building instance segmentation and evaluated representative YOLO-based segmentation models, Mask R-CNN, and detection-only RT-DETR baselines under consistent experimental settings. The benchmark covered detection accuracy, mask quality, inference efficiency, model complexity, convergence behavior, cross-dataset transferability, and controlled-degradation robustness.

The experimental results show that YOLO-based segmentation models provide competitive performance for building instance segmentation in the evaluated WHU and Inria settings. Among the compared mask-predicting models, YOLOv11x-seg achieved the strongest or near-strongest mask-level accuracy and is suitable for accuracy-oriented applications. YOLO26x-seg showed strong detection coverage and competitive mask performance, while YOLOv11m-seg provided a favorable balance among accuracy, inference speed, and model complexity, making it a practical candidate for efficiency-oriented deployment.

The additional cross-dataset and degradation experiments further indicate that high-capacity YOLO-seg models can recover competitive performance after target-domain training, but direct zero-shot transfer from WHU to Inria remains challenging. The controlled degradation analysis shows that YOLOv11x-seg is relatively robust to shadow/occlusion but more sensitive to Gaussian blur, suggesting that image quality and target-domain adaptation remain important for reliable operational deployment.

Overall, the proposed benchmark provides quantitative and practical reference evidence for selecting building instance segmentation models under different remote sensing application requirements. Future work will extend this benchmark to more datasets, imaging conditions, degradation types, and multimodal remote sensing data, and will further investigate lightweight and boundary-aware instance segmentation models for large-scale geospatial applications.

## Figures and Tables

**Table 1:** Manual quality check of generated instance annotations.

Subset	Checked Images	Checked Instances	Correct Instances	Merged Adjacent Buildings	Over-Separated Fragments	Invalid Polygons
Train	180	180	177	1	1	0
Validation	60	60	58	1	0	1
Test	60	60	56	1	2	1
Total	300	300	292	3	3	2

**Table 2:** Comparison of detection and segmentation performance of different models on the WHU dataset.

Model	P(B)	R(B)	mAP@0.5 (B)%	mAP@0.5:0.95 (B)%	P(M)	R(M)	mAP@0.5(M)%	mAP@0.5:0.95(M)%	mIoU
YOLOv8n-seg	0.916	0.854	93.101	75.338	0.909	0.836	91.144	66.510	0.845
YOLOv8m-seg	0.917	0.879	94.764	80.046	**0.916**	0.865	93.489	71.617	0.844
YOLOv8l-seg	**0.919**	0.882	94.873	80.485	0.914	0.869	93.405	72.118	0.834
YOLOv8x-seg	0.916	0.880	94.723	80.301	**0.916**	0.873	93.992	72.898	0.846
YOLOv11n-seg	0.915	0.850	92.954	75.070	0.911	0.836	91.304	66.117	0.836
YOLOv11m-seg	0.916	0.881	94.711	80.009	0.914	0.873	93.837	72.573	0.801
YOLOv11l-seg	0.915	0.880	94.916	80.279	0.911	0.873	93.813	72.621	0.856
YOLOv11x-seg	0.917	0.881	94.940	80.679	**0.916**	**0.875**	**94.210**	**72.931**	**0.877**
YOLO26n-seg	0.895	0.832	91.898	73.884	0.893	0.818	90.478	65.746	0.843
YOLO26m-seg	0.905	0.873	94.658	79.736	0.904	0.865	93.700	72.094	0.843
YOLO26l-seg	0.905	0.876	94.703	79.648	0.903	0.867	93.647	71.851	0.852
YOLO26x-seg	0.911	**0.884**	**95.157**	**80.808**	0.909	**0.875**	94.128	72.883	0.849
Mask R-CNN	0.911	0.743	91.030	69.200	0.908	0.739	90.450	67.890	0.823
RT-DETR-l	0.903	0.868	92.459	75.529	-	-	-	-	-
RT-DETR-x	0.900	0.845	90.035	72.554	-	-	-	-	-

Note: RT-DETR-l and RT-DETR-x were included only as auxiliary detection-only Transformer baselines. Since they do not output instance masks in the implemented setting, mask-based metrics, including P(M), R(M), mAP(M), and mIoU, were not computed and are denoted as “-“. Bold values indicate the best performance among the compared models for the corresponding metric.

**Table 3:** Statistical reliability analysis of representative models over three independent random-seed runs.

Model	mAP@0.5(M)%	mAP@0.5:0.95(M)%	mIoU	mAP@0.5:0.95(B)%
YOLOv8x-seg	93.992 ± 0.011	72.898 ± 0.007	0.846 ± 0.002	80.301 ± 0.009
YOLOv11x-seg	94.210 ± 0.008	72.931 ± 0.004	0.877 ± 0.001	80.679 ± 0.008
YOLO26x-seg	94.128 ± 0.009	72.883 ± 0.003	0.849 ± 0.002	80.808 ± 0.006

**Table 4:** Comparison of inference efficiency and complexity across models.

Model	Time(ms)	FPS	Params(M)	GFLOPs@512
YOLOv8n-seg	7.72	129.5	3410	7.772
YOLOv8m-seg	9.48	105.5	27.286	67.190
YOLOv8l-seg	10.92	91.6	45.998	135.083
YOLOv8x-seg	11.81	84.6	71.828	210.687
YOLOv11n-seg	9.49	105.3	2.877	6.336
YOLOv11m-seg	11.77	85	22.421	72.866
YOLOv11l-seg	15.82	63.2	27.678	85.081
YOLOv11x-seg	16.39	61	62.143	190.423
YOLO26n-seg	10.9	91.7	3.126	6.726
YOLO26m-seg	12.36	80.9	27.112	84.828
YOLO26l-seg	15.98	62.6	31.516	96.604
YOLO26x-seg	16.92	59.1	70.694	216.104
MaskR-CNN	29.4	34.01	43.918	87.843
RT-DETR-l	31.06	32.2	32.97	70.581
RT-DETR-x	33.7	29.7	67.468	150.159

**Table 5:** Late-stage fluctuation analysis of representative segmentation models over the final 20 recorded epochs.

Model	Epoch	P(M)	R(M)	mAP@0.5(M)	mAP@0.5:0.95(M)	Val/Box Loss	Val/Seg Loss
YOLOv8x-seg	93–112	0.003014	0.003303	0.000992	0.002444	0.001939	0.011454
YOLOv11x-seg	101–120	0.002334	0.002281	0.000578	0.002101	0.002605	0.015859
YOLO26x-seg	101–120	0.002555	0.002334	0.002514	0.002327	0.002421	0.013019

**Table 6:** Summary of multi-dimensional performance comparison of representative models.

Model	Key Accuracy (M)	mIoU	FPS	Advantages	Limitations	Overall
YOLOv11x-seg	**94.210/72.931**	**0.877**	61.0	Highest mask AP in this benchmark; favorable boundary quality	High cost	**Accuracy-oriented candidate**
YOLO26x-seg	94.128/72.883	0.849	59.1	High recall; strong coverage	Slightly lower precision	**Coverage-oriented candidate**
YOLOv11m-seg	93.837/72.573	0.801	**85.0**	Favorable accuracy–speed–cost balance	Lower peak accuracy	**Balanced candidate**
YOLOv8x-seg	93.992/72.898	0.846	84.6	Stable baseline	Slightly weaker in complex scenes	Competitive baseline

Note: Bold values indicate the best performance among the compared models for the corresponding metric.

**Table 7:** Inria in-domain evaluation of representative YOLO-seg models under different initialization strategies.

Model	Init Strategy	mAP@0.5(M)	P(M)	R(M)
YOLOv8x-seg	WHU-Init	0.817	0.844	0.765
YOLOv8x-seg	COCO-Init	0.809	0.844	0.763
YOLOv11x-seg	WHU-Init	0.819	0.849	0.765
YOLOv11x-seg	COCO-Init	**0.821**	**0.851**	**0.768**
YOLO26x-seg	WHU-Init	0.814	0.838	0.757
YOLO26x-seg	COCO-Init	0.819	0.848	0.761

Note: Bold values indicate the best performance among the compared models for the corresponding metric.

**Table 8:** Controlled-degradation robustness analysis of YOLOv11x-seg under shadow/occlusion and Gaussian blur conditions.

Test Condition	mAP@0.5(B)	mAP@0.5(M)	P(M)	R(M)	Relative Drop
Clean Baseline	0.8363	0.8168	0.8483	0.7632	0.00%
Shadow/Occlusion	0.8217	0.8023	0.8372	0.7494	−1.78%
Gaussian Blur	0.7300	0.7066	0.7821	0.6550	−13.50%

## Data Availability

We used the publicly available WHU Building dataset. The URL is https://gpcv.whu.edu.cn/data (accessed on 9 March 2026).

## Abbreviations

The following abbreviations are used in this manuscript:

CNN	Convolutional Neural Network
FCN	Fully Convolutional Network
ViT	Visual Transformers
FPN	Feature Pyramid Networks
MSEIES	Multi-scale Edge Information Enhancement and Selection Module
DEIEM	Dual Edge Information Fusion Module
DenseFPN	Dense Feature Pyramid Network
SCP	Spatial Context Pyramid
YOLO	You Only Look Once
RPN	Region Proposal Network
RoI	Region of Interest
PSDA	Pyramid Space Deformable Aggregation
HFSA-Unet	Hybrid First- and Second-Order Attention Network
P	Precision
R	Recall
FPS	Frames Per Second
mIoU	Mean Intersection Over Union
GFLOPs	Giga Floating-point Operations Per Second
GT	Ground Truth
